# *Fusobacterium* Species and Subspecies Differentially Affect the Composition and Architecture of Supra- and Subgingival Biofilms Models

**DOI:** 10.3389/fmicb.2019.01716

**Published:** 2019-07-30

**Authors:** Thomas Thurnheer, Lamprini Karygianni, Manuela Flury, Georgios N. Belibasakis

**Affiliations:** ^1^Division of Oral Microbiology and Immunology, Clinic of Conservative and Preventive Dentistry, Center of Dental Medicine, University of Zurich, Zurich, Switzerland; ^2^Division of Oral Diseases, Department of Dental Medicine, Karolinska Institutet, Huddinge, Sweden

**Keywords:** *Fusobacterium nucleatum*, subspecies, supra- and subgingival biofilm models, bacterial growth, fluorescent *in situ* hybridization, confocal laser scanning microscopy

## Abstract

Fusobacteria are common obligately anaerobic Gram-negative bacteria of the oral cavity that may act as a bridge between early and late colonizing bacteria in dental plaque and have a role in oral and extra-oral infections. *Fusobacterium nucleatum* has a crucial role in oral biofilm structure and ecology, as revealed in experimental and clinical biofilm models. The aim of this study was to investigate the impact of various *Fusobacterium* species on *in vitro* biofilm formation and structure in three different oral biofilm models namely a supragingival, a supragingival “feeding”, and a subgingival biofilm model. The standard six-species supragingival and “feeding” biofilm models employed contained *Actinomyces oris*, *Candida albicans*, *Streptococcus mutans*, *Streptococcus oralis*, *Veillonella dispar*, and *Fusobacterium* sp. The subgingival biofilm model contained 10 species (*A. oris*, *Campylobacter rectus*, *F. nucleatum* ssp. *nucleatum*, *Porphyromonas gingivalis*, *Prevotella intermedia*, *Streptococcus anginosus*, *S. oralis*, *Tannerella forsythia*, *Treponema denticola*, and *V. dispar*). Six different *Fusobacterium* species or subspecies, respectively, were tested namely *F. nucleatum* ssp. *fusiforme*, *F. nucleatum* ssp. *nucleatum*, *F. nucleatum* ssp. *polymorphum*, *F. nucleatum* ssp. *vincentii*, *F. naviforme*, and *F. periodonticum*). Biofilms were grown anaerobically on hydroxyapatite disks in 24-well culture dishes. After 64 h, biofilms were either harvested and quantified by culture analysis or proceeded to fluorescent *in situ* hybridization (FISH) and confocal laser scanning microscopy (CLSM). All *Fusobacterium* species tested established well in the biofilms, with CFUs ranging from 1.4E+04 (*F. nucleatum* ssp. *fusiforme*) to 5.6E+06 (*F. nucleatum* ssp. *nucleatum*). The presence of specific *Fusobacterium* sp./ssp. induced a significant decrease in *C. albicans* levels in the supragingival model and in *V. dispar* levels in the “feeding” supragingival model. In the subgingival model, the counts of *A. oris*, *S. oralis*, *P. intermedia*, *P. gingivalis*, and *C. rectus* significantly decreased in the presence of specific *Fusobacterium* sp./ssp. Collectively, this study showed variations in the growing capacities of different fusobacteria within biofilms, affecting the growth of surrounding species and potentially the biofilm architecture. Hence, clinical or experimental studies need to differentiate between *Fusobacterium* sp./ssp., as their biological properties may well vary.

## Introduction

The disposition of biofilms, which are composed of dynamic substratum-attached microbial networks, is an everyday phenomenon in nature (Hall-Stoodley et al., 2004; Marsh, 2005). In particular, the oral cavity is an excellent aquatic ecosystem allowing for the settlement of myriads of microorganisms on the salivary pellicle-coated tooth surfaces and epithelial layers of gingiva (Kolenbrander et al., 2002; Marsh, 2004). In fact, more than 700 different bacterial species embedded in extracellular polysaccharide-affluent matrix account for the formation of the multispecies oral communities (Foster and Kolenbrander, 2004; Keijser et al., 2008). The latter encounter 1,000-times more resistance to antimicrobial agents; host immunity; nutrient restriction; alternating oxygen-, nitrogen-, and carbon dioxide supply; and abrupt temperature fluctuation (Davies, 2003; Welin-Neilands and Svensater, 2007; Marsh and Devine, 2011).

Selective salivary-derived proteins allow for the initial adherence of the early bacterial colonizers, namely streptococci and actinomyces, upon direct adsorption onto the host surfaces (Takahashi and Nyvad, 2011; Nikitkova et al., 2013). The succession of intra-generic and intra-species microbial partnerships within the dental plaque biofilms is thereby promoted by co-adhesion of planktonic to immobilized microorganisms or co-aggregation among distinct bacterial species (Katharios-Lanwermeyer et al., 2014; Palmer, 2014). Thereafter, the maturation of oral biofilms is activated by the extracellular polymeric substances (EPS) formation and is mediated by *Fusobacterium nucleatum*, a Gram-negative microorganism, which is located in the interface of early and late colonizers such as obligate anaerobes and streptococci (He et al., 2012). Indeed, streptococci are found to prevail the first 6–48 h, whereas *F. nucleatum*-mediated coaggregation takes place after the first 48 h of oral biofilm formation (Dige et al., 2007).

*F. nucleatum* can be considered as a periodontal pathogen, due to its enhanced prevalence within the subgingival biofilm, which is retrieved from deep periodontal pockets (Gursoy et al., 2008). Specifically, *F. nucleatum* triggers the production of matrix metalloproteinases by the host contributing initially to periodontal inflammation and then to irreversible periodontal disease (Gursoy et al., 2010). Interestingly, *F. nucleatum* also consists of an adhesin-rich outer membrane enabling the adhesion to various salivary proteins, other microorganisms, and host substrata (Kaplan et al., 2009; Coppenhagen-Glazer et al., 2015). Additional virulence properties associated with *F. nucleatum* include its enhanced hemolytic activity, and the production of hydrogen sulfide (H_2_S) serves as key virulence traits employed by *F. nucleatum* (Gaetti-Jardim Júnior and Avila-Campos, 1999; Okamoto et al., 2000; Yoshida et al., 2009). *F. nucleatum* can also invade and reside within the human gastrointestinal tract (Strauss et al., 2011).

Due to the possession of numerous virulence properties, *F. nucleatum* may have an integral role in regulating oral biofilm formation and subsequent growth. Interestingly, an earlier report confirmed this assumption by revealing a significant decrease in the total colony forming units (CFU) of an *in vitro* supragingival biofilm model in the absence of *F. nucleatum* (Guggenheim et al., 2001a; Thurnheer et al., 2003). Reversely, the absence of streptococci or, streptococci and actinomyces altogether, from an *in vitro* subgingival biofilm model resulted in a less compact and more dispersed distribution of *F. nucleatum* within the biofilm mass (Ammann et al., 2013), confirming a structural functional interrelationship with the other species. Yet, given the importance of *F. nucleatum* as an essential “bridging” biofilm component, there is to date no evidence in literature how different *Fusobacterium* species can affect the formation and structure of multispecies oral biofilms *in vitro*. Moreover, to our knowledge, there is at present restricted evidence in literature of potential differences between *F. nucleatum* subspecies (closely related fusobacterial species) in the context of biofilm maturation. Therefore, six different *Fusobacterium* species or subspecies, namely *F. nucleatum* ssp. *fusiforme*, *F. nucleatum* ssp. *nucleatum*, *F. nucleatum* ssp. *polymorphum*, *F. nucleatum* ssp. *vincentii*, *F. naviforme*, and *F. periodonticum* were tested for their ability to integrate into and affect the growth of three different *in vitro* oral biofilm models (standard supragingival, supragingival “feeding” and subgingival), respectively. The null hypothesis of this study was that specific *Fusobacterium* species have no significant impact on the overall growth of *in vitro* oral biofilms and can all act as a bridge between their surrounding bacterial partners promoting biofilm maturation.

## Materials and Methods

### *In vitro* Biofilm Experiments

For this study, three different *in vitro* biofilm models were used namely the standard supragingival model, the supragingival “feeding” model, and the subgingival model. The procedures for biofilm production have been described in detail before (Shapiro et al., 2002; Ammann et al., 2012; Thurnheer et al., 2014b). In brief, the standard supragingival biofilm employed contained *Actinomyces oris* (OMZ 745), *Candida albicans* (OMZ 110), *F. nucleatum* ssp. *nucleatum* (OMZ 598), *Streptococcus oralis* SK 248 (OMZ 607), *Streptococcus mutans* (OMZ 918), and *Veillonella dispar* ATCC 17748^T^ (OMZ 493). Six different inocula were used: as a control, the inoculum containing the six strains above was used, whereas in each of the further five inocula, one of the following *Fusobacterium* strains was used: (1) *F. nucleatum* ssp. *fusiforme* ATCC 51190 (OMZ 642), (2) *F. nucleatum* ssp. *vincentii* ATCC 49256^T^ (OMZ 635), (3) *F. nucleatum* ssp. *polymorphum* ATCC 10953 (OMZ 595), (4) *Fusobacterium naviforme* (*formerly F. nucleatum* ssp*. naviforme*) NCTC 11464 (OMZ 594), and (5) *Fusobacterium periodonticum* ATCC 33693^T^ (OMZ 636). The taxonomic standing of the used fusobacteria strains as well as the phylogenetic distances among them has previously been reported using monoclonal antibodies, ribotyping, and 16S rRNA sequencing, highlighting the individuality of the species (Thurnheer et al., 1999; Gmür et al., 2006).

Biofilms were grown anaerobically in 24-well polystyrene cell culture plates on hydroxyapatite disks that had been preconditioned for pellicle formation in whole un-stimulated pooled saliva (in the following termed saliva) for 4 h. To initiate a biofilm experiment, disks were covered for the first 16 h with 1.6 ml of growth medium containing 70% saliva, 30% modified fluid universal medium (mFUM; Guggenheim et al., 2001a) supplemented with Sørensen’s buffer (final pH 7.2), and 200 μl of a cell suspension prepared from equal volumes and densities of each strain. The medium was changed after 16 and 40 h. For the first 16 h, the medium contained 0.3% glucose. After 16 h, the medium was replenished with one containing 0.15% glucose and 0.15% sucrose, instead of 0.3% glucose. In order to remove non-adherent microorganisms, biofilms were dipped three times in saline after 16, 20, and 24 h as well as after 40, 44, and 48 h. After 64 h of incubation, the biofilms were dip-washed again and either harvested for culture analyses by vigorous vortexing in 1 ml of 0.9% NaCl or proceeded to staining and confocal laser scanning microscopy (CLSM) (see below).

The supragingival “feeding” model in the following termed “feeding” model was established in order to mimic more accurately the fast and feast periods experienced by natural dental plaque (Thurnheer et al., 2006). Therefore, the standard experimental protocol described above was modified as follows: (1) the proportion of saliva and mFUM was reversed to 30% saliva and 70% mFUM and (2) exposure to this altered medium was time limited. That is, after inoculation the disks remained for only 45 min in the feeding solution containing 0.3% glucose. Thereafter, they were subjected to three consecutive 1-min washes in 2 ml 0.9% NaCl to remove growth medium and free-floating cells but not bacteria adhering firmly to HA disks. The biofilms were then further incubated in new wells containing 1.6 ml of saliva and no mFUM. Only after 16, 20, 24, 40, 44, and 48 h were biofilms pulse-fed by transferring the disks for 45 min into 30% saliva/70% mFUM with 0.15% glucose and 0.15% sucrose. Thereafter, they were washed as described above and re-incubated in saliva. Fresh saliva was provided after 16 and 40 h. After 64 h, biofilms were washed and processed for further analyses.

In order to grow subgingival *in vitro* biofilms, the protocol for standard supragingival biofilms described above was modified as follows: (1) 10 species were used instead of six, namely *A. oris* (OMZ 745), *Campylobacter rectus* (OMZ 388), *F. nucleatum* ssp. *nucleatum* (OMZ 598), *Porphyromonas gingivalis* ATCC 33277^T^ (OMZ 925), *Prevotella intermedia* ATCC 25611^T^ (OMZ 278), *Streptococcus anginosus* ATCC 9895 (OMZ 871), *S. oralis* SK 248 (OMZ 607), *Tannerella forsythia* (OMZ 1047), *Treponema denticola* ATCC 35405^T^ (OMZ 661), and *V. dispar* ATCC 17748^T^ (OMZ 493) and (2) the growth medium contained 60% saliva, 10% fetal bovine serum, and 30% FUM. To generate subgingival biofilms, the same procedure as described for the standard supragingival biofilm model was applied.

Total CFU, streptococci, and other taxa were assessed by culture using selective and non-selective media (Guggenheim et al., 2001a; Van Der Ploeg and Guggenheim, 2004; Thurnheer et al., 2014a). CFU data are not provided for *T. denticola* and *T. forsythia*, since these species do not grow on solid agar. Therefore, the CFU-related data provided are on the remaining eight species in the “subgingival” biofilm.

### Staining of Biofilms

Biofilms were stained by fluorescence *in situ* hybridization following earlier described protocols (Thurnheer et al., 2004; Ammann et al., 2012). In brief, prehybridization (15 min, 46°C) was performed in 500 μl hybridization buffer with 40% formamide in the absence of any oligonucleotide probes. Thereafter, 500 μl of hybridization buffer (40% formamide) was used for each biofilm, supplemented with the genus-specific Cy3-labeled probe STR405 (5′-TAGCCGTCCCTTTCTGGT-3′) and Cy5-labeled probe FUS664 (5′-CTTGTAGTTCCGCYTACCTC-3′) to stain streptococci and fusobacteria, respectively, at a concentration of 20 ng/μl. The incubation time for the hybridization was at least 3 h at 46°C in the dark. After the incubation, biofilms were transferred into washing buffer preheated to 48°C and incubated for 20 min at this temperature. For counterstaining, biofilms were stained using a mixture of 3 μM YoPro 1 iodide (Invitrogen, Carlsbad, CA) and 15 μM Sytox green (Invitrogen; for 20 min, at room temperature in the dark), following the fluorescence *in situ* hybridization procedure. After staining, the samples were embedded upside down on chamber slides in 100 μl of Mowiol (Guggenheim et al., 2001b).

### Confocal Laser Scanning Microscopy (CLSM) and Image Analysis

Stained biofilms were examined by confocal laser scanning microscopy using a Leica TCS SP5 microscope (Leica Microsystems, Wetzlar, Germany) with ×100/1.4 NA oil-immersion objective lens, in conjunction with an argon laser at 488 nm excitation, a DPSS diode laser at 561 nm, and a Helium-Neon laser at 633 nm excitation. Filters were set to 500–540 nm for YoPro/Sytox, to 570–600 nm for Cy3, and to 660–710 nm for Cy5, respectively. Biofilms were scanned in sequential mode, and z-series were generated by vertical optical sectioning using a step size of 1 μm. Image acquisition was done in ×8 line average mode, and scans were recombined and processed using IMARIS 7.6.5 software (Bitplane, Zurich, Switzerland), without any qualitative changes to the raw images.

### Statistical Analyses

Three individual experiments were performed and each group represented in triplicate biofilm cultures per experiment. A two-way analysis of variance in conjunction with Tukey’s multiple comparison text was used to evaluate the differences between the control and each experimental group. The significance level was set to *p* < 0.05. Values below the assay’s detection limit were ascribed the lowest detection limit value to allow for logarithmic transformation. The Prism v.7.0 statistical analysis software (GraphPad, La Jolla, CA, USA) was used to analyze the data.

## Results

**Figure 1:**
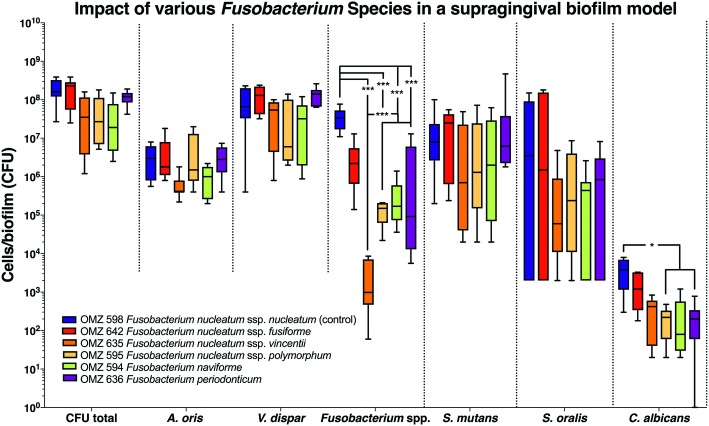
Fusobacterial growth in a supragingival biofilm model. Colony forming units (CFUs) of the supragingival biofilm model (*A. oris*, *F. nucleatum*, *S. mutans*, *S. oralis*, *V. dispar*, and *C. albicans*). The different colors correspond to experimental groups of different fusobacteria used, as indicated. Data derive from nine independent experiments in which every group was represented in triplicate biofilm cultures. Box plots represent the CFUs determined by selective agar plating, while horizontal lines indicate their median values. Undetectable values were ascribed the lowest detection limit value of the assay to allow for log transformation. Asterisks (*) represent significant difference compared with the control group (*p* < 0.05). Statistically significant differences compared with the control group are indicated with asterisks (*p < 0.05; ***p < 0.001).

A total of six *Fusobacterium* species/subspecies were tested in the three different experimental biofilm model variants. In the 70:30 model, the total bacterial numbers (CFU) yielded after the completion of the biofilm culture were similar irrespective of the *Fusobacterium* spp. used. Accordingly, no significant differences were observed between experimental groups among the other bacterial species in the biofilms (Figure 1). Nevertheless, the number of fusobacteria in the biofilm significantly differed, depending on the species or subspecies incorporated. In particular, levels of *F. nucleatum* ssp. *vincenti*, *F. nucleatum* ssp. *polymorphum*, *F. naviforme*, as well as that of *F. periodonticum* were significantly lower in the biofilm than the *F. nucleatum* ssp. *nucleatum* control, which is used in the standard biofilm model (Figure 1). The least incorporated in terms of numbers within the biofilm proved to be *F. nucleatum* ssp. *vincentii*. The numbers of *C. albicans* were also differentially affected according to the subspecies present in the biofilm, depending on the used fusobacteria, with a significant reduction in the presence of *F. nucleatum* ssp. *polymorphum*, *F. naviforme*, and *F. periodonticum*, compared to the *F. nucleatum* ssp. *nucleatum* control.

**Figure 2:**
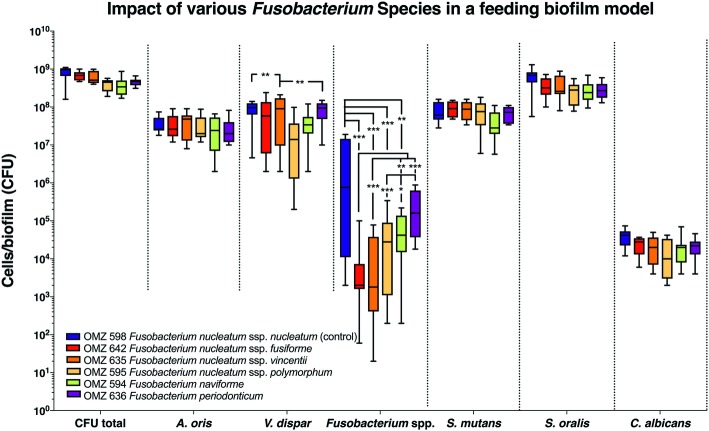
Fusobacterial growth in a supragingival “feeding” biofilm model. Colony forming units (CFUs) of the supragingival “feeding” biofilm model (*A. oris*, *F. nucleatum*, *S. mutans*, *S. oralis*, *V. dispar*, and *C. albicans*). The different colors correspond to experimental groups of different fusobacteria used, as indicated. Data derive from nine independent experiments in which every group was represented in triplicate biofilm cultures. Box plots represent the CFUs determined by selective agar plating, while horizontal lines indicate their median values. Undetectable values were ascribed the lowest detection limit value of the assay to allow for log transformation. Asterisks (*) represent significant difference compared with the control group (*p* < 0.05). Statistically significant differences compared with the control group are indicated with asterisks (*p < 0.05; **p < 0.01; ***p < 0.001).

Next, the “feeding” model was investigated (Figure 2). The total bacterial numbers in the biofilm culture were similar, irrespective of the *Fusobacterium* ssp. used. Accordingly, no significant differences were detected between experimental groups among the other bacterial species or *C. albicans* in the biofilms (Figure 2). Apart from *F. periodonticum*, all other *F. nucleatum* ssp. were significantly reduced, compared to the ssp. *nucleatum* control. Interestingly, significant variations in numbers existed also among the different *F. nucleatum* ssp., indicating different incorporation capacities according to subspecies in this model. Among the other species in the biofilm, variations also existed in the numbers of *V. dispar*.

**Figure 3:**
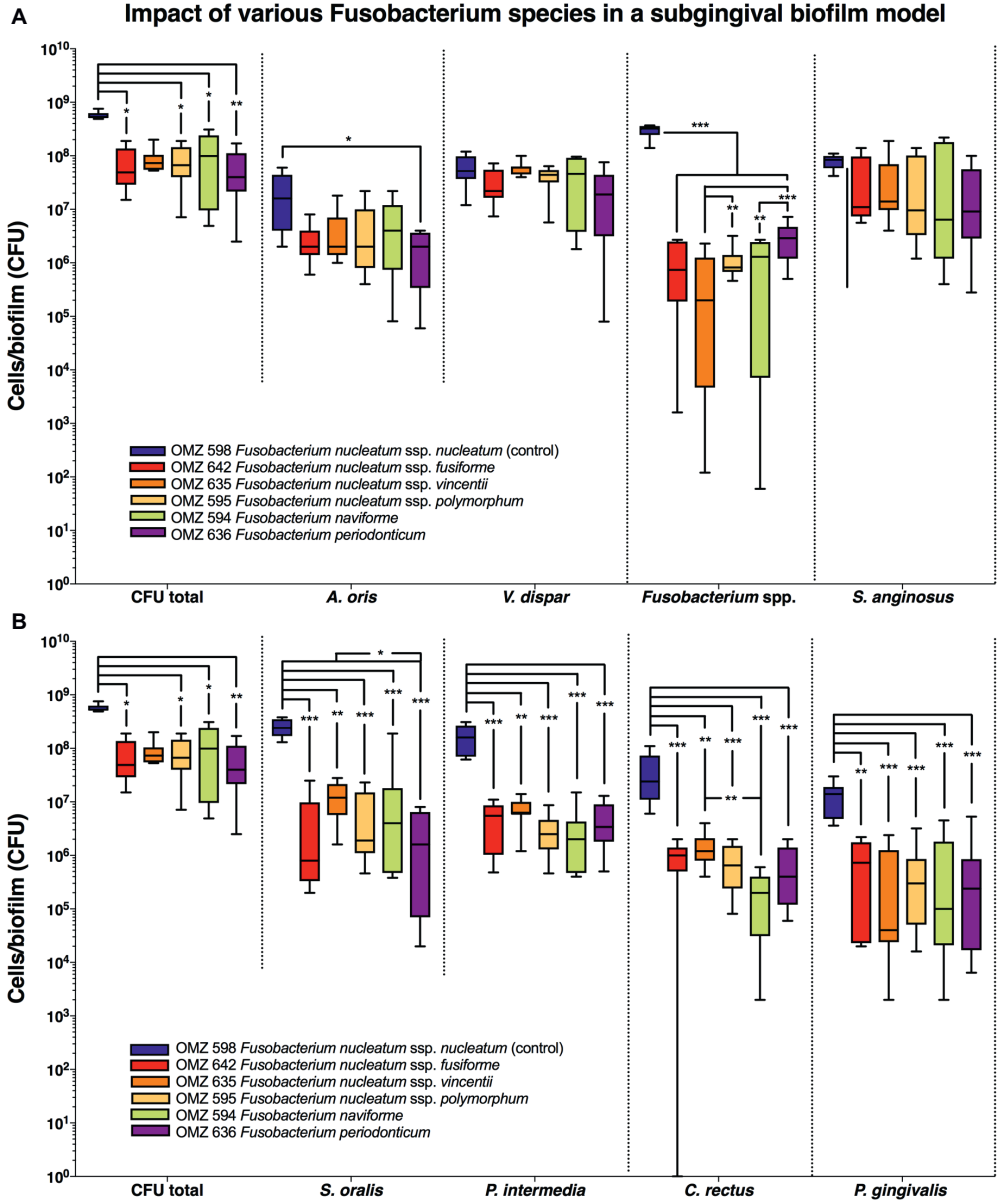
Fusobacterial growth in a subgingival biofilm model. Colony forming units (CFUs) of the subgingival biofilm model (*A. oris*, *C. rectus*, *F. nucleatum*, *P. intermedia*, *P. gingivalis*, *S. anginosus*, *S. oralis*, *T. denticola*, *T. forsythia*, and *V. dispar*). The total CFUs are provided in both **(A)** and **(B)**, in order to facilitate their comparative assessment to all individual species. The different colors correspond to experimental groups of different fusobacteria used, as indicated. Data derive from nine independent experiments in which every group was represented in triplicate biofilm cultures. CFU data are not provided for *T. denticola* and *T. forsythia*, since these species do not grow on solid agar. Box plots represent the CFUs determined by selective agar plating, while horizontal lines indicate their median values. Undetectable values were ascribed the lowest detection limit value of the assay to allow for log transformation. Asterisks (*) represent significant difference compared with the control group (*p* < 0.05). Statistically significant differences compared with the control group are indicated with asterisks (*p < 0.05; **p < 0.01; ***p < 0.001).

In the “subgingival” model, the total bacterial numbers in the biofilm were significantly lower in the case of all *Fusobacterium* ssp. tested than the *F. nucleatum* control (Figure 3A). Accordingly, the numbers of all other *Fusobacterium* ssp. tested were also lower than the *F. nucleatum* control group, while significant variations also existed between the subspecies. A reduction in *A. oris* was also observed with all *F. nucleatum* ssp. tested but proved to be significant only in the case of *F. periodonticum* (Figure 3A). Significant reductions were also observed in the numbers of *P. gingivalis*, *S. oralis*, *P. intermedia*, and *C. rectus* with all *F. nucleatum* ssp. tested, compared to the *F. nucleatum* control (Figure 3B).

**Figure 4:**
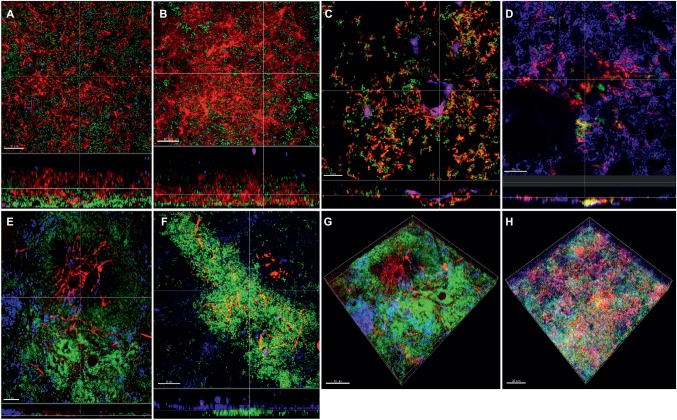
Incorporation of various *Fusobacterium* species in the supragingival biofilm model. Panels **(A–F)** depict confocal laser scanning microscopic (CLSM) image stack, while panels **(G)** and **(H)** represent 3D reconstructions of *in vitro* supragingival biofilms. Due to FISH staining, fusobacteria appear red and streptococci blue, and due to DNA staining with Sytox/YoPro 1, bacteria appear green. **(A)** *F. nucleatum* ssp*. nucleatum* (control), **(B)** *F. nucleatum* ssp*. fusiforme*, **(C)** *F. nucleatum* ssp*. vincentii*, **(D)** *F. nucleatum* ssp*. polymorphum*, **(E)** *F. periodonticum*, **(F)** *F. naviforme*, **(G)** *F. periodonticum* (3D), and **(H)** *F. nucleatum* ssp*. fusiforme* (3D); scale bars: 10 μm.

Apart from the effect of the different subspecies on microbial numbers in the biofilms, their localization pattern within the biofilm was investigated by CLSM (Figure 4). In the supragingival biofilm model, a tighter distribution of *F. nucleatum* ssp. *fusiforme* was observed (Figures 4B,H), compared to *F. nucleatum* ssp. *nucleatum* control (Figures 4A,E), with a comparable distribution of streptococci. In case of *F. nucleatum* ssp. *vincentii* (Figure 4C) and *F. nucleatum* ssp. *polymorphum* (Figure 4D), the distribution of both fusobacteria and streptococci appeared to be sparser than the control. The distribution of *F. periodonticum* was characterized by more distinctive own cell clusters in the mass of the biofilm (Figure 4G).

**Figure 5:**
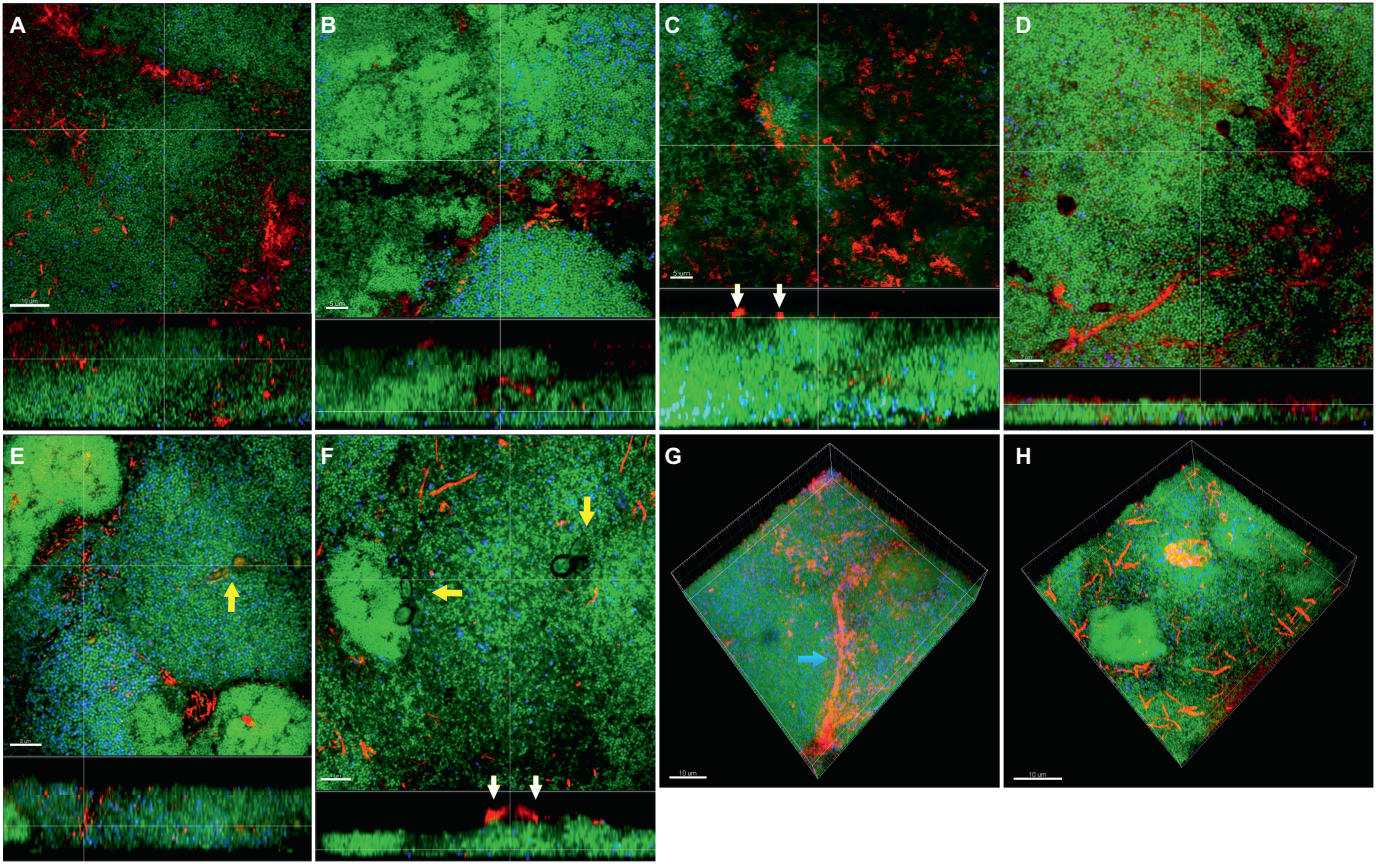
Incorporation of various *Fusobacterium* species in the feeding biofilm model. Panels **(A–F)** depict confocal laser scanning microscopic (CLSM) image stack, while panels **(G)** and **(H)** represent 3D reconstructions of *in vitro* “feeding” supragingival biofilms. Due to FISH staining, fusobacteria appear red and streptococci blue, and due to DNA staining with Sytox/YoPro 1, bacteria appear green. **(A)** *F. nucleatum* ssp*. nucleatum* (control), **(B)** *F. nucleatum* ssp*. fusiforme*, **(C)** *F. nucleatum* ssp*. vincentii*, **(D)** *F. nucleatum* ssp*. polymorphum*, **(E)** *F. periodonticum*, **(F)** *F. naviforme*, **(G)** *F. nucleatum* ssp*. polymorphum* (3D), and **(H)** *F. periodonticum* (3D); white arrows indicate the localization of fusobacteria [*F. nucleatum* ssp*. vincentii* and *F. naviforme*, panels **(C)** and **(F)**, respectively] near the biofilm substratum interface, yellow arrows indicate the presence of *C. albicans*, and the blue arrow shows the filamentous structure resulting from the deposition of multiple *F. nucleatum* ssp. *vincentii* cells. Scale bars: 5–10 μm.

In the “feeding” model, the distribution of fusobacteria was comparable between *F. nucleatum* ssp. *nucleatum* (Figures 5A,E), *fusiforme*, (Figure 5B), *vincentii* (Figure 5C), *F. naviforme* (Figure 5F) and *F. periodonticum* (Figure 5G), with scattered aggregate distribution through the biofilm and streptococcal clusters occasionally identified in close proximity. The distribution of *F. nucleatum* ssp*. polymorphum* (Figure 5D) displayed a more characteristic filamentous structure resulting from the deposition of multiple fusobacteria across the biofilm mass (Figures 5G,H).

**Figure 6:**
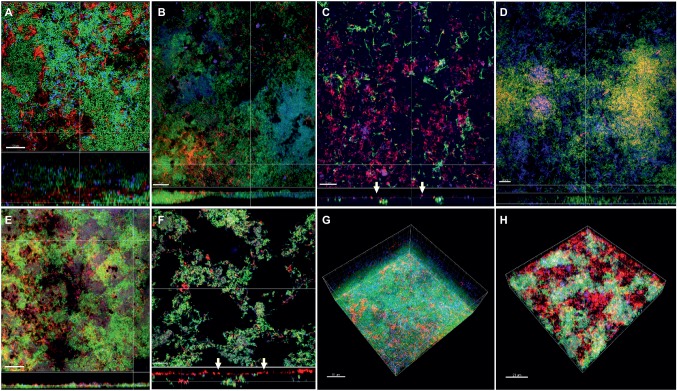
Incorporation of various *Fusobacterium* species in the subgingival biofilm model. Panels **(A–F)** depict confocal laser scanning microscopic (CLSM) image stack, while panels **(G)** and **(H)** represent 3D reconstructions of *in vitro* subgingival biofilms. Due to FISH staining, fusobacteria appear red and streptococci blue, and due to DNA staining with Sytox/YoPro 1, bacteria appear green. **(A)** *F. nucleatum* ssp*. nucleatum* (control), **(B)** *F. nucleatum* ssp*. fusiforme*, **(C)** *F. nucleatum* ssp*. vincentii*, **(D)** *F. nucleatum* ssp*. polymorphum*, **(E)** *F. periodonticum*, **(F)** *F. naviforme*, **(G)** *F. nucleatum* ssp*. nucleatum* (3D), and **(H)** *F. naviforme* (3D); white arrows indicate the localization of fusobacteria [*F. nucleatum* ssp*. vincentii* and *F. naviforme*, panels **(C)** and **(F)**, respectively] near the biofilm substratum interface. Scale bars: 5–10 μm.

In the subgingival biofilm model, the distribution of *F. nucleatum* ssp. *nucleatum* (Figures 6A,E), *polymorphum* (Figures 6D,G), and *F. periodonticum* (Figures 6F,H) was scattered through the biofilm mass along with streptococci, whereas in the case of *fusiforme* (Figure 6B) and *vincentii* (Figure 6C), their distribution was also scattered, but the proximity with streptococci was less pronounced.

## Discussion

Fusobacteria are crucial microbial constituents of dental biofilms, with a crucial role as “bridging” microorganisms between early and late colonizing species (Bradshaw et al., 1998; Henne et al., 2018; Brennan and Garrett, 2019). *F. nucleatum* can communicate with other bacterial species in the biofilm *via* quorum-sensing signaling molecules (Frias et al., 2001), such as autoinducer-2 (Jang et al., 2013a), which has the capacity to regulate the interaction with other bacteria (Jang et al., 2013b). Apart for its potential involvement in periodontal disease, there is increasing evidence of extra-oral translocation of this species and association with extra-oral infections and systemic conditions (Han and Wang, 2013), to the extent that it is recently being considered as a potential oncobacterium (Brennan and Garrett, 2019). It can be classified in several subspecies (Jousimies-Somer, 1997), and it is not clear whether they display different metabolic versatility on a given microenvironmental niche, such as the supragingival or the subgingival biofilm environment (Rogers, 1998). Based on genomic evidence, it was recently proposed that *F. nucleatum* subsp. *nucleatum*, subsp. *polymorphum*, subsp. *vincentii*, and subsp. *animalis* should be classified as *F. nucleatum*, *F. polymorphum*, *F. vincentii*, and *F. animalis*, respectively (Kook et al., 2017). While two of the strains used in the present study, namely *F. nucleatum* ssp*. fusiforme* OMZ 642 (ATCC 51190) and *F. nucleatum* ssp*. vincentii* OMZ 635 (ATCC 49256), have been reported to have high genotypic similarity to a degree that they could be classified as a single subspecies (Kook et al., 2013), in our earlier work using ribotyping, antigenicity, and 16S rRNA sequencing, they are shown to be phenotypically and phylogenetically of adequate distance (Thurnheer et al., 1999; Gmür et al., 2006). An earlier study also developed a specialized qPCR assay in order to distinguish between these two subspecies species (annotated as *F. nucleatum* subsp. *vincentii* ATCC 49256 and subsp. *fusiforme* ATCC 51190(T), respectively; Shin et al., 2010, indicating that minute genomic differences may exist.

There is evidence that different *F. nucleatum* subspecies may differentially affect neutrophil function and the oxidative killing by neutrophils (Kurgan et al., 2017) denoting variations in their virulence properties. Yet, potential differences in their functional properties within biofilms have not yet been elucidated. The present study has addressed this issue by using an *in vitro* biofilm experimental approach and demonstrates differential biofilm behavior among the different subspecies. These may also be influenced by micro-ecological conditions, as portrayed by the three different biofilm models used here (i.e., “supragingival,” “feeding” and “subgingival”). Collectively, *F. nucleatum* ssp. *nucleatum* was the most well-adapted subspecies in the present *in vitro* biofilm models used, irrespective of their “supragingival” or “subgingival” profile. This is evident by the consistently higher numbers and evenly scattered distribution in the biofilm, compared to the other subspecies tested. The least favorably adapted subspecies in terms of numbers in the biofilm appeared to be *vincentii*, whereas the one behaving closest to *F. nucleatum* ssp. *nucleatum* appeared to be the species *F. periodonticum*. More detailed comparisons of their genomes and identification of genomic variations to ssp. *nucleatum* may help understand better their properties and capacities to adapt to different environmental pressures. It is worth further investigating the interkingdom interaction between *C. albicans* and *F. nucleatum* ssp. *polymorphum*, or *F. naviforme* and *F. periodonticum* species, as this yeast displayed reduced numbers in the “supragingival model.” Indeed, interaction between *C. albicans* and *F. nucleatum* may lead to a mutual attenuation of their virulence, resulting in a potential commensalism (Bor et al., 2016). *C. albicans* has been also shown to support the presence of strictly anaerobic bacteria under oxygen-rich conditions, including fusobacteria, in early *in vitro* biofilms (Janus et al., 2017), denoting its influence on the bacteriome in oral biofilms.

In the “feeding” oral biofilm model, variations were observed in the numbers of *V. dispar* depending on the fusobacterial species or subspecies used. As the exposure to nutritional constituents in this model deviates from that of the standard “supragingival” biofilm model, this difference may denote variations in the metabolic queues between *V. dispar* and fusobacterial subspecies, depending on the environmental exposures. It was recently shown that, *via* its catalase, *Veillonella* sp. can rescue the growth of *F. nucleatum* under microaerophilic conditions or the presence of *S. gordonii* (Zhou et al., 2017). *S. gordonii* has also displayed strong coaggregation properties to *F. nucleatum* (Mutha et al., 2018). While *S. gordonii* was not present in the model used here, *S. mutans* exposed to sugar may have altered the micro-environmental conditions in the biofilm, potentially affecting the interaction of *F. nucleatum* ssp. and *V. dispar*. It is worth investigating further the differential interactions of various *F. nucleatum* ssp. with *V. dispar*, which may account for the differences with the growth patterns observed in the present study. In regard to the biological significance of the intercommunication between fusobacteria and *S. mutans*, a recent study revealed that binding of *F. nucleatum* ssp. *polymorphum* to *S. mutans* can be attributed to recognition of the *F. nucleatum* ssp. *polymorphum* adhesin RadD by the *S. mutans* adhesin SpaP (Guo et al., 2017). Thus, the RaD/SpaP adhesin pair serves as a binding mechanism for fusobacteria and implies their high virulence potential, since *S. mutans* interacts with only a limited number of other bacterial species (Wang et al., 2011).

In the subgingival biofilm model, the periodontal pathogens *P. gingivalis*, *P. intermedia*, and *C. rectus* proved to grow better in the presence of *F. nucleatum* ssp. *nucleatum* than the other subspecies tested. This may imply that this subspecies is better adapted to co-exist synergistically with other periodontal pathogens in a biofilm environment. However, it could also reflect differences in the growth profile of the various *F. nucleatum* ssp. themselves, as they also proved to grow at lower numbers in the biofilm than the *F. nucleatum* ssp. *nucleatum* control. Since there are no studies describing oral fusobacterial interactions with other periodontal pathogens on a subspecies level in humans (Antiabong et al., 2013), we can only speculate that *F. nucleatum* ssp. *nucleatum* can bind to periopathogens such as *P. gingivalis*, *P. intermedia*, and *C. rectus* due to the presence of specific pairs of multifactorial proteins, which are not associated with other *F. nucleatum* subspecies.

Interestingly, a reduction in numbers of *S. oralis*, a commensal microorganism, was also observed. We have shown earlier that *F. nucleatum* growth was significantly enhanced following the late addition of the streptococci when compared to their absence (Ammann et al., 2013). Others have also identified interaction networks between *F. nucleatum* and *Streptococcus mitis* (Zhang et al., 2019), or *P. gingivalis* (Mohammed et al., 2017). Nevertheless, variations depending on the subspecies of *F. nucleatum* have been rarely taken under consideration.

In conclusion, differences in the growth and localization patters of *F. nucleatum* in biofilms may be observed based on the subspecies. Subspecies other than *nucleatum*, or species *F. periodonticum*, appear to be less efficiently growing in the biofilm and exhibit a more condensed localization pattern. Differences in the genotypic, phenotypic, and metabolic properties among fusobacteria may account for their different behavioral patterns. It is therefore important to consider further and define more accurately fusobacteria at the subspecies level, in order to understand their role not only in biofilm-associated oral diseases but also in light of their involvement in extra-oral infections and malignancies.

## Data Availability

All datasets generated for this study are included in the manuscript.

## Author Contributions

TT conceived the idea for this manuscript. MF conducted the experiments. LK was involved in the data analysis. GB was involved in study design, data interpretation, and manuscript drafting. All authors critically reviewed the manuscript and approved its final version.

### Conflict of Interest Statement

The authors declare that the research was conducted in the absence of any commercial or financial relationships that could be construed as a potential conflict of interest.
